# Mandatory health insurance and health care utilization in Togo

**DOI:** 10.1186/s12913-022-08942-y

**Published:** 2022-12-14

**Authors:** Dossè Mawussi Djahini-Afawoubo, Segnon T. Aguey

**Affiliations:** Department of Economics, The University of Lomé, PO Box: 1515, Lomé, Togo

**Keywords:** Health insurance, Health care utilization, Togo

## Abstract

**Background:**

Despite the implementation of a mandatory health insurance (MHI) scheme in Togo since 2011, its coverage rate remains low, resulting in a high out-of-pocket payment rate. More than 10 years after its implementation, there are few empirical studies investigating the extent to which Togo’s mandatory health insurance has improved beneficiaries’ access to health care. Examining how MHI and healthcare use in Togo are related is the goal of this study.

**Methods:**

We use data from the Harmonized Survey on Living Conditions of Households (EHCVM), conducted in the member states of the West African Economic and Monetary Union (WAEMU) in 2018–2019 and covering 6,171 households in Togo. We employ multinomial logistic regression, given that the dependent variable is polytomous.

**Results:**

The results reveal a high rate of non-utilization of healthcare professionals in the case of illness, even among individuals with MHI coverage. Furthermore, the findings show that the MHI increases the likelihood of seeing a specialist physician and other formal health care professionals when sick. The results also reveal that a household’s wealth is positively correlated with the likelihood of seeing formal health care professionals. Urban residents are statistically and significantly more likely than rural residents to see both a specialist physician and a general practitioner. The Grand Lomé region has a statistically and significantly higher likelihood of seeing a specialist physician than the Maritime region.

**Conclusion:**

The results support the government’s plan to implement universal health insurance. The government should take action to raise the standard of treatment provided to insured patients in health care centers. Additionally, the government should consider waiving medical fees for low-income policyholders. When waiving medical costs for low-income policyholders, the Togolese government should focus on the regions with the worst economic conditions. These interventions should be essential to ensure that no one is left behind. The difference between urban and rural communities should be reduced through supply-side policies that focus on rural areas.

## Introduction

Economic theory emphasizes the importance of health for the economic development of nations. Endogenous growth theory [1, 2] argues that differences in economic growth between countries stem mainly from differences in investment in human capital, especially in education, health, and nutrition. Recognizing the benefits of good health for economic growth, a number of developing nations have put in place health insurance programs to increase access to care by lowering out-of-pocket expenses. Several studies have demonstrated the positive effect of health insurance on health care utilization [3–8]. Hou and Chao [4] find health insurance has increased beneficiaries’ utilization of acute surgeries and inpatient services in Georgia. Camacho and Conover [5] find that mothers with health insurance have better access to health facilities, while Miller et al. [3] find that health insurance has increased the use of preventive services in Colombia. Lobato, Carpio, and Klein [6] find that health insurance has increased the probability of seeing a doctor, receiving medication, and receiving medical analysis in Peru. Palmer et al. [7] find a positive impact of health insurance on inpatient and outpatient visits in Vietnam. In India, Fan, Karan, and Mahal [8] find that health insurance significantly reduces out-of-pocket inpatient expenditures.

Togo, the focus of this study, is a West African country, a member of the West African Economic and Monetary Union (WAEMU), which is classified as one of the least developed countries [9]. Togo has a surface area of approximately 56,600 km^2^ and a population of nearly 7,886,000 inhabitants in 2001 [10]. According to the National Institute of Statistics, Economic Studies, and Demography (INSEED), at the national level, 45.5% of the population lives below the poverty line, with location-based inequalities (58.8% in rural areas compared to 26.5% in urban areas) and regional inequalities as well. The Savannah region has the highest poverty incidence (65.1%), while the Grand Lomé region has the lowest poverty incidence (22.3%), a difference of nearly 43% points in absolute terms [11].

Aware of the positive effects improving health, in its anti-poverty strategy, the Togolese government has identified access to health care as an important component. The government has therefore implemented, since 2011, a mandatory health insurance scheme (MHI) for state employees and their dependents [9]. Almost ten years after its implementation, there are surprisingly few empirical studies [9, 12, 13] on Togo’s mandatory health insurance. Djahini-Afawoubo and Atake [9] estimate the willingness-to-pay (WTP) of informal sector workers (ISW) to have access to the MHI and analyze the main determinants of WTP. Atake and Amendah [13] examine the incidence and intensity of catastrophic health expenditures among households insured by the MHI. To the best of our knowledge, only Atake [12] has investigated the extent to which the MHI has improved access to health care for beneficiaries by assessing whether the utilization of health services, choice of provider, and financial protection due to health insurance significantly differ by health insurance type. However, by focusing on insured households in a single region, both residence-based inequalities and regional inequalities are ignored. Yet, given the global commitment to leave no one behind, analyzing residence-based inequalities and regional inequalities may provide useful insight to policymakers. In contrast to Atake [12], this study uses the most recent nationally representative database on household living conditions in Togo to assess the relationship between the MHI and the likelihood of seeing health care professionals in case of illness. Therefore, this paper accounts for both residence-based inequalities and regional inequalities. The findings of this study may help decision-makers in Togo, in particular, and in sub-Saharan Africa, in general, implement universal health insurance coverage.

A brief overview of the Togolese health insurance scheme is presented in the following section. The third section discusses the empirical strategy and presents the data source, while the fourth section presents the results. Section five discusses the policy implications, and the last section concludes the paper.

### Overview of the mandatory health insurance scheme in Togo

Since 2011, Togo has implemented the MHI scheme for civil servants, civil servant retirees, and up to six of their family members. The objective of the Togolese health insurance scheme is to provide quality healthcare as well as financial protection to registered households by covering risks related to illness, non-occupational accidents, and maternity. The scheme is managed by the National Health Insurance Institute (INAM). It is financed by monthly contributions amounting to 7% of the insured’s basic salary. Half of this contribution is paid by the insured (3.5%), and the other half is paid by the state [9, 13]. According to Wright et al. [14], INAM reimburses facilities up to 80% of the official reimbursement rate for public sector facilities, whereas the remaining 20% (or higher) is paid by the insured member to the facility. INAM contracted with public and private health facilities, pharmacies, and eye-care facilities. Covered treatments include those for cardiovascular illnesses, metabolic and endocrine diseases, nephrological diseases, systemic diseases, rheumatoid conditions, mental illnesses, bowel disease, cancer, malignancies of the lymphatic tissue, hematomas, ophthalmic diseases, minor and major surgeries, dental care, and diseases of the ear, nose, and throat. General medicine, specialty consultations, prenatal consultations, and certain drugs and medical devices are also covered. Consultations with informal health care providers are not covered by the MHI [15]. More than 10 years after its implementation, the coverage rate of the MHI remains low. The effective coverage, considering all people of working age, is estimated at around 4% [9] of the country’s population, and the share of the MHI was estimated at 3.5% of current health expenditures (CHE) in 2019 [16]. Furthermore, private health insurance penetration is less than 2% of the Togolese population [14, 17]. In 2019, the share of voluntary health insurance (VHI) is estimated at 6.2% of CHE [16]. This situation results in a high rate of out-of-pocket payments (OOP), estimated at 66.2% of CHE in 2019 [16].

## Materials and methods

### Empirical strategy to estimate the correlation between MHI and health care utilization

To estimate the correlation between MHI and health care utilization, we employ a multinomial logistic model, given that the dependent variable is polytomous [18, 19], consisting of 5 categories that have no natural ordering. We use the following equation:

1 $${Y}_{i}={\alpha }_{0}+{\alpha }_{1}{MHI}_{i}+{X\alpha }_{2}+{\mu }_{i}$$

Where ${Y}_{i}$ is the type of health care professional the individual *i* saw in the case of illness. MHI is a binary variable, taking 1 if the individual *i* has access to MHI and 0 if he or she is uninsured. *X* is a matrix of j individual and household level characteristics with their respective coefficients that can influence *Y*. ${\mu }_{i}$ captures all the remaining variation with $\mu_i\;IIDN(0,\delta^2)$). Assuming *MHI* is exogenous, then a multinomial logistic regression of Eq. (1) will produce consistent estimates for *Y*. It follows that, the coefficient of interest ${\alpha }_{1}$ can be regarded as the true impact of MHI on *Y* [20]. Given that the MHI is a compulsory scheme with no risk for self-selection, we believe that the assumption of exogeneity of the MHI variable is likely.

### Description of data

The data underlying the findings of this study are extracted from the database of the Harmonized Survey on Living Conditions of Households, conducted in the member states of the West African Economic and Monetary Union. This database contains the information needed to monitor and evaluate poverty and household living conditions in each of the member countries. In Togo, the INSEED implemented the EHCVM in 2018–2019, with support from the World Bank and the WAEMU Commission. The EHCVM is a nationally representative survey of 6,171 households, which are representative of the geopolitical zones (Grand Lomé, Maritime, Plateau, Central, Kara, and Savannah), at both the urban and rural levels [21]. The survey used two main instruments: a household/individual questionnaire and a community-level questionnaire. We used information from the household questionnaire. This household questionnaire was administered to all household members. The survey was conducted by CAPI (Computer-Assisted Personal Interview) using smartphones. The responses were entered in real time on the smartphones. The interviews were conducted face-to-face [22]. Our study population for assessing the effect of the MHI coverage is all household members. Only those who responded “yes” to the following query were included in the study: “In the previous 30 days, was [NAME] sick?” Overall, our final sample consisted of 8,419 individuals after removing all observations that were not relevant to this study. Malaria/fever (59%), stomach problems (11%), as well as colds and coughs (5.6%), were the most common illnesses reported. Diarrhea, accidents or injuries, dental problems, skin problems, eye problems, blood pressure, typhoid fever, diabetes, meningitis, and sore throats are among the other illnesses reported (see Table 1). All of these illnesses are covered by the MHI if treated at formal, accredited health centers [15].

**Table 1:** Frequency of type of illnesses reported by the sampled individuals

Type of illnesses	Number of individuals	Percentage
Fever/Malaria	4,951	58.81
Diarrhea	215	2.55
Accident/Injury	263	3.12
Dental problems	82	0.97
Skin problems	241	2.86
Eye diseases	128	1.52
Tension problem	210	2.49
Typhoidal fever	32	0.38
Stomach trouble	920	10.93
Sore throats	45	0.53
Coughs and colds	475	5.64
Diabetes	24	0.29
Meningitis	4	0.05
Other	829	9.85
Total	8,419	100.00

### Dependent variable

The dependent variable is the type of health care professional seen in the case of illness. The following question from the survey is used: “Who did [NAME] first see this episode of illness?” The possible answers are formal health care professionals (such as a specialist physician, a general physician, a dental doctor, and other formal healthcare professionals), and informal health care providers such as traditional healers, marabouts, or others. We code 0 if the individual did not see a health care professional, 1 if he or she saw a specialist physician, 2 if he or she saw a general practitioner, 3 if he or she saw a dental doctor, 4 if he or she saw other formal health care professionals and 5 if he or she saw informal health care providers. Prior to the analysis of the data, we dropped all observations where the type of health care professional seen in the case of illness was “a dental doctor,” because there were too few observations for this outcome.

### Explanatory variables

The selection of the explanatory variables follows Andersen’s behavioral model of health care utilization, which posits that health care utilization is a function of three major elements: predisposing factors (socio-demographic factors), enabling factors (e.g., income and health insurance), and health care needs such as functional disability and chronic illnesses [23–25]. The study’s main explanatory variable is health insurance status. Health insurance is supposed to have a positive effect on health care utilization through reduced out-of-pocket payments. Several studies show that health insurance has a positive effect on health care utilization [20, 23, 24]. To measure health insurance status, we use the following questions from the survey: “Does [NAME] have health insurance coverage?“ and “Who sponsors or funds [NAME]’s health insurance?“ Does [NAME] have another sponsor or source of funding that needs to be specified? Based on the individual’s responses to these questions, we code 1 if the answer is either “state/program” or “state employer,“ 2 if the answer is “private,“ “private employer,“ or “other,“ and 0 if the individual is uninsured. We dropped all observations where health insurance status was private, private employer, or other. Based on the answers to the following question: “Does [NAME] have another sponsor or source of funding to be specified?“ we notice that only four individuals have specified another sponsor or source of funding. To avoid including in our analysis individuals with complementary health insurance, we dropped all these observations where the individual specified another sponsor or source of funding. Finally, health insurance status equals 1 if the individual is covered by MHI, and health insurance status equals 0 for uninsured individuals. Besides health insurance status, we include the wealth indicator as an enabling factor [20, 25]. Given the importance of the informal sector in Togo [9], it is difficult to obtain reliable data on income and wealth. Following [26], we measure a household’s wealth by a composite variable using factor analysis consisting of a household’s ownership of durable goods (radio, television, motorbike, bike, car, refrigerator, fan, air conditioner, latrine, agricultural land, a residential building or dwelling (other than the one occupied by the household), and roof materials. The choice of these assets is motivated by data availability. The wealth variable ranges from 0, where the household has none of the items, to 1, where the household has all the items. The study controls for predisposing factors such as the level of education of the household head, gender of the household head, location area, age group of the individual, region of residence, the size of the household, and the marital status of the household head. Several studies have also used these variables [12, 20, 25]. Finally, hospitalization in the past 12 months prior to the survey is used as a proxy for the health care needs factors [25]. All variables used in the study are described in Table 2.

**Table 2:** Description of main variables of the study

Variables	Description
Insurance status	Dichotomous variable. 0. Uninsured; 1. MHI
Type of healthcare professional visited in case of illness	Polytomous variable. 0. Did not visit; 1. The individual saw a specialist physician; 2. The individual saw a general physician; 3. The individual saw a dental doctor; 4. The individual saw other formal healthcare professionals; 5. The individual saw informal healthcare professionals.
Wealth	Index of wealth, based on durable goods ownership (radio, television, motorbike, bike, car, refrigerator, fan, air conditioner latrine) and roof materials. The wealth variable ranges from 0, where the household has none of the items, to 1, where the household has all the items
Education of household head	Polytomous variable. 0. No formal education; 1. Primary; 2. Secondary; 3. University
Location	Location area. Dichotomous variable 1. Urban; 0. Rural
Sex	Sex of household head. Dichotomous variable. 1. Male; 0. Female
Household size	Continuous variable. Number of all individuals in the household
Age	The age group of individuals is polytomous. 0. Under 18 years; 1. 18–35; 2. 35–45; 3. 45–55; 4. 55–65; 5. 65–75; 6. More than 75
Marital status	The marital status of the household head is polytomous. 1. Single; 2. Married; 3. Widow or widower; 4. Divorced
Hospitalization	Hospitalization status in the last 12 months prior to the survey. Dichotomous variable. 1. Yes; 0. No

Prior to the econometric analysis, we dropped all observations where the type of health care professional seen in the case of illness is “dental doctor”, because there are too few observations for this outcome.

## Results

### Descriptive statistics

Table 3 presents descriptive statistics on the coverage of the MHI scheme and health care utilization according to individuals’ socio-demographic characteristics. At the national level, only 4.2% of respondents who have been sick during the last 30 days are covered by the MHI scheme. Individuals whose household heads have a university degree have the highest rate of MHI coverage (18.8%). The rate of MHI coverage among individuals whose household heads have a secondary education level is 7.1%, compared to 3.7% among individuals whose household heads have no formal education. Women (4.1%) and men (4.4%) have almost equal MHI coverage rates, while there are significant disparities between the country’s administrative regions. The Kara region has the highest MHI coverage rate (6.2%), while the Central region exhibits the lowest rate (2.8%). By area of residence, the coverage of the MHI is more than twice as high for urban residents (6.8%) compared to rural residents (2.9%). The seniors over 75 years old (1.7%) have the lowest MHI coverage rate.

We also note some disparities regarding the type of health care professional seen in the case of illness. Overall, nearly the half of respondents (49.8%) did not see a health care professional in the case of illness. Only 3.4% of respondents saw a specialist physician, while 15.1% saw a general practitioner. The results show that 34% of individuals with the MHI coverage did not see a health care professional in the case of illness, compared to approximately 51% of uninsured individuals. The Savannah region, one of the poorest regions of the country, has the highest rate of not seeing a health professional in the case of illness (54%), while the Grand Lomé region has the lowest rate (45%). The Grand Lomé region has the highest rate of seeing a general practitioner (26.7%) as well as the highest rate of seeing a specialist (10.1%). The central region, in contrast, has the lowest rates (9.2% for general practitioner consultations and 2% for specialist consultations).

**Table 3:** MHI coverage rate (%) and health care utilization (%) among individuals who have been sick during the last 30 days by sociodemographic characteristics

Socio-demographic characteristics	MHI	Uninsured	Generalist	Specialist	Other formal health care professional	Informal health care providers	Nonuse of health care professional in the case of illness	Number of observations
**Gender**								
Female	4.1	95.9	15.5	3.4	3.2	1.2	49.7	4,474
Male	4.4	95.6	14.7	3.4	30.8	1.2	49.9	3,945
**Region**								
Lomé	5.1	94.9	26.7	10.1	17.7	0.5	45.0	1,168
Maritime	2.7	97.3	17.7	2.5	31.1	0.7	48.0	1,308
Plateau	3.1	96.9	12.1	2.3	27.9	0.9	56.8	169
Centrale	2.8	97.2	9.2	2.0	41.2	3.0	44.6	1,062
Kara	6.2	93.8	13.6	2.2	36.4	1.8	46.0	1,351
Savannah	4.9	95.1	13.3	2.4	29.9	0.6	53.8	1,84
**Age**								
Under 18 years	4.4	95.6	14.9	3.1	31.0	1.1	49.9	3,862
18–35	3.7	96.3	16.5	3.9	32.2	1.3	46.1	1,937
35–45	5.6	94.4	14.5	2.6	30.1	1.2	51.6	853
45–55	3.9	96.1	15.8	3.7	28.1	1.5	50.9	590
55–65	5.6	94.4	12.4	3.3	30.1	0.8	53.4	395
65–75	3.0	97.0	14.0	5.5	25.0	2.5	53.0	200
More than 75	1.7	98.3	15.0	4.0	26.1	0.5	54.4	582
**Education**								
No formal education	3.7	96.3	14.5	2.9	30.2	1.3	51.1	5,739
Primary	2.5	97.5	13.9	3.5	31.8	0.7	50.1	1,303
Secondary	7.1	92.9	18.0	5.3	30.0	1.2	45.5	1,276
University	18.8	81.2	31.7	8.9	27.7	0.0	31.7	101
**Location area**								
Urban	6.8	93.2	22.9	6.4	24.4	0.7	45.6	2,703
Rural	2.9	97.1	11.5	2.0	33.3	1.4	51.8	5,716
**Insurance status**								
MHI			18.1	11.3	35.7	0.9	34.0	353
Uninsured			15.0	3.0	30.2	1.2	50.6	8,066
**National**	4.2	95.8	15.1	3.4	30.5	1.2	49.8	8,419
Number of observations	353	8,066	1,275	285	2,565	98	4,196	

Table 4 reports descriptive statistics on the reason for not seeing a health care professional in the case of illness. Overall, self-medication (57.5%) is the major reason respondents did not see any health care professional when they were sick in the last 30 days prior to the survey. This is followed by the lack of money (33%), and the fact that the respondent does not consider it necessary (5.6%) to see a health care professional. Distance is mentioned by only 1.1% of respondents as a major reason for not seeing a health care professional when they were sick in the last 30 days. The results show that the proportion of respondents who mention the lack of money as the most important reason for not seeing a health care professional in the case of illness increases as we move from a high level of education to a lower level of education of the household head. Indeed, 9.4% of household heads with university education give a lack of money as the major reason for not seeing a health care professional, compared to 29% of household heads with secondary education, 35.5% of household heads with primary education, and 33.5% of household heads with no formal education. The lack of money is also mentioned by 10% of respondents with MHI coverage, compared to 33.7% of uninsured respondents. The results show that, the proportion of respondents who reports the lack of money as the major reason for not seeing a health care professional in the case of illness is almost twice as high for the Kara region (44.3%) compared to the Grand Lomé region (21.3%).

**Table 4:** Reason for not seeing a healthcare professional among individuals who did not see a doctor when they were sick in the past 30 days by socio-demographic characteristics (%)

Socio-demographic characteristics	Not necessary	Too expensive	Too far	Self-medication	Lack of money	Other	Number of observations
**Gende**r							
Female	5.1	0.9	1.2	57.4	33.3	2.1	2,226
Male	6.1	0.8	1.0	57.6	32.7	1.8	1,97
**Region**							
Lomé	5.9	0.2	0.0	70.3	21.3	2.3	526
Maritime	5.4	0.5	1.0	46.6	43.2	3.3	627
Plateau	6.9	2.0	1.6	66.0	22.4	1.1	959
Centrale	7.4	0.4	2.5	46.4	41.6	1.7	474
Kara	5.2	0.6	1.6	46.5	44.3	1.8	621
Savannah	3.6	0.6	0.3	61.6	32.0	1.9	989
**Age**							
Under 18 years	5.5	0.8	1.2	58.9	31.5	2.1	1,928
18–35	5.8	0.8	0.8	60.5	30.3	1.8	894
35–45	7.3	0.9	1.1	59.1	30.7	0.9	440
45–55	4.3	0.7	0.7	55.7	36.3	2.3	300
55–65	4.2	1.0	1.0	53.1	38.4	2.3	211
65–75	4.7	0.0	0.9	40.6	52.8	1.0	106
More than 75	5.4	1.3	1.9	48.6	40.0	2.8	317
**Education**							
No formal education	5.1	0.9	1.3	57.5	33.5	1.7	2,931
Primary	6.3	0.5	0.8	54.0	35.5	2.9	653
Secondary	7.2	1.0	0.5	60.3	29.0	2	580
University	6.2	0.0	0.0	81.3	9.4	3.1	32
**Location area**							
Urban	7.5	1.4	0.0	65.3	23.4	2.4	1,233
Rural	4.8	0.6	1.6	54.3	37.0	1.7	2,963
**Health insurance status**							
MHI	7.5	0.0	0.0	77.5	10.0	5.0	120
Uninsured	5.5	0.9	1.1	56.9	33.7	1.9	4,076
Total	5.6	0.8	1.1	57.5	33.0	2.0	4,196
Number of observations	234	35	46	2,413	1,386	82	

### Determinants of health care utilization

In this section, we analyze factors associated with health care utilization using multinomial logistic regression. Table 4 reports the findings. The Likelihood Ratio (LR) test shows that the null hypothesis that all regression coefficients are jointly equal to zero is rejected at the 1% significance level. This implies that the variables included in the regressions create a significant improvement in the fit of the model, suggesting a good fit [18]. We examine the variance inflation factors (VIF) to check for multicollinearity in our sample. The largest VIF is 2.5, and the mean VIF is 1.7, which does not suggest high multicollinearity [12]. Finally, we employ the Hosmer-Lemeshow test to assess goodness-of-fit, which is satisfactory since we cannot reject the null hypothesis (Table 5) that the fitted model is the correct model and the sample is sufficiently large [27].

The results show that MHI coverage is positively correlated with the likelihood of seeing a specialist physician in the case of illness, rather than not seeing any health care professional. The same is true for the probability of seeing other formal health care professionals. However, the correlation between the MHI and the likelihood of seeing a general practitioner in the case of illness is not statistically significant. The correlation between the MHI and the probability of seeing informal health care providers in the case of illness is negative but not statistically significant. All else being equal, the likelihood of seeing a specialist physician rather than not seeing any health care professional in the case of illness increases by 4.6% points (at the 1% significance level) when the individual is covered by the MHI compared to an uninsured individual. Similarly, all else being equal, the chances of seeing other formal health care professionals in the case of illness increase by nearly 7.6% points (at the 5% significance level) when the individual is covered by the MHI compared to an uninsured individual.

The results reveal a positive and statistically significant correlation between household wealth and the probability of seeing formal health care professionals in the case of illness. All else being equal, an instant increase in the wealth index results in an increase in the likelihood of seeing a specialist physician in the case of illness by 6.3% points (at the 1% significance level). Similarly, the chances of seeing a general practitioner and other formal health care professionals increase by 17.3 and 23.4% points, respectively.

Being hospitalized in the previous 12 months prior to the survey, according to the findings, is positively correlated with the likelihood of seeing formal health care professionals, rather than not seeing any health care professional in the case of illness. All else being equal, the likelihood of seeing a specialist physician rather than not seeing any health care professional in the case of illness increases by 5.4% points (at the 1% significance level) when the individual has been hospitalized in the last 12 months preceding the survey, compared to an individual who has not been hospitalized. Similarly, the chances of seeing a general practitioner increase by 6.3% points, all else being equal. The chances of seeing other health care professionals also increase by 5.2% points, but only at the 10% significance level.

The findings also show that, all else equal, urban residents have 5.8% points more chances than rural residents to see a general practitioner when sick. In contrast, urban residents have 8.4% points fewer chances than rural residents to see other formal health care professionals. The findings indicate that the likelihood of seeing a specialist physician is higher in the Grand Lomé region compared to the Maritime region, all else being equal. The likelihood of seeing a general practitioner rather than not seeing any health care professional in the case of illness is statistically and significantly lower in all other regions (except the Grand Lomé region) compared to the Maritime region. We find that the gender of the household head has no statistically significant effect on the likelihood of seeing formal health care professionals. The size of the household is negatively correlated with the probability of seeing a specialist in the case of illness, but only at the 10% significance level.

**Table 5:** Results of the multinomial logistic regressions for the determinants of healthcare utilization in Togo

VARIABLES	The individual saw a specialist	The individual saw a general practitioner	The individual saw other formal health care professionals	The individual saw informal health care providers
Type of health insurance = 1, MHI	0.0462***	0.00404	0.0758**	0.00114
	(0.0148)	(0.0224)	(0.0336)	(0.00655)
Wealth index	0.0627***	0.173***	0.234***	-0.0141
	(0.0150)	(0.0435)	(0.0642)	(0.0133)
Level of education of household head = Primary school (the reference is no formal education)	-0.00356	-0.0135	0.0388**	0.00119
	(0.00499)	(0.0129)	(0.0178)	(0.00324)
Level of education of household head = Secondary school	-0.00538	-0.0168	0.0375**	-0.00150
	(0.00416)	(0.0121)	(0.0180)	(0.00311)
Level of education of household head = University	-0.0109	0.0463	-0.0292	0.00418
	(0.00736)	(0.0381)	(0.0513)	(0.0143)
Sex of household head = Male (the reference is female)	0.00227	-0.00315	-0.0163	-0.000640
	(0.00368)	(0.00964)	(0.0128)	(0.00226)
Location area = Urban (the reference is rural)	0.00154	0.0578***	-0.0842***	-0.00629*
	(0.00540)	(0.0142)	(0.0172)	(0.00333)
Age group = 18–35 (the reference is under 18 years)	0.000264	0.0139	-0.0388*	0.000575
	(0.00591)	(0.0149)	(0.0212)	(0.00337)
Age group = 35–45	-0.0114	0.00290	-0.0507*	0.0101
	(0.00697)	(0.0201)	(0.0283)	(0.00645)
Age group = 45–55	-0.0125*	-0.00910	-0.0949***	-0.00197
	(0.00752)	(0.0216)	(0.0294)	(0.00446)
Age group = 55–65	-0.00359	-0.00347	-0.0759**	-0.00224
	(0.00954)	(0.0242)	(0.0329)	(0.00494)
Age group = 65–75	-0.00216	0.00279	-0.0751*	0.0275*
	(0.0125)	(0.0320)	(0.0412)	(0.0162)
Age group = More than 75 years	0.00246	-0.0115	-0.0790***	0.00104
	(0.00967)	(0.0212)	(0.0302)	(0.00537)
Region = Plateau (the reference group is Maritime)	-0.000649	-0.0636***	-0.0262	-0.000423
	(0.00590)	(0.0169)	(0.0206)	(0.00316)
Region = Central	-0.00483	-0.0948***	0.0799***	0.0183***
	(0.00629)	(0.0175)	(0.0242)	(0.00611)
Region = Kara	9.45e-05	-0.0564***	0.0694***	0.0101**
	(0.00630)	(0.0178)	(0.0226)	(0.00464)
Region = Savannah	0.00378	-0.0392**	-0.0153	-0.00158
	(0.00639)	(0.0178)	(0.0211)	(0.00302)
Region = Grand Lomé	0.0375***	0.000800	-0.107***	0.00480
	(0.0115)	(0.0209)	(0.0237)	(0.00689)
Household size	-0.00115*	-0.00131	-0.000299	0.000509
	(0.000691)	(0.00167)	(0.00210)	(0.000322)
Marital status = Married (the reference group is single)	0.000748	0.0163	0.0543***	0.00106
	(0.00500)	(0.0138)	(0.0195)	(0.00399)
Marital status = Widow or widower	0.0121	0.0118	0.0250	-0.00380
	(0.0114)	(0.0235)	(0.0314)	(0.00455)
Marital status = Divorced	-0.00874	0.0481	-0.0203	-0.00699*
	(0.00847)	(0.0302)	(0.0354)	(0.00420)
Hospitalization in the last 12 months = yes (the reference group is no)	0.0537***	0.0631***	0.0518*	0.00176
	(0.0141)	(0.0234)	(0.0279)	(0.00521)
Hosmer-Lemeshow test				
chi2	26.2			
Prob > chi2	0.76			
Multicollinearity				
Largest VIF	2.5			
Mean VIF	1.7			
Likelihood Ratio test				
LR chi2(92)	650.1			
Prob > chi2	0.000			
Pseudo R2	0.05			
Observations	5,868	5,868	5,868	5,868

Standard errors in parentheses; *** *p* < 0.01, ** *p* < 0.05, * *p* < 0.1; the reference category for the dependent variable is 0: did not see any health care professional in case of illness. The reduction in the number of observations is due to missing data.

## Discussions

This study analyzes the relationship between the Togolese MHI and health care utilization. The findings show that the MHI significantly increases the probability of seeing a specialist physician and other formal health care professionals such as nurses and care assistants in the case of illness rather than not seeing any health care professional. The findings also show that household wealth is positively correlated with the likelihood of seeing formal healthcare professionals (specialist physicians, general practitioners, and other formal healthcare professionals), as opposed to not seeing any health care professional. These results corroborate Andersen’s behavioral model. MHI coverage and household wealth are key enablers of health-care utilization [28–30]. These results are consistent with the literature [20, 23, 31–33]. Allin et al. [31] find a positive effect of wealth on health care utilization in 12 countries. Agbanyo and Peprah [20] also find that a household’s wealth is a strong predictor of the use of institutional health facilities for delivery among expectant mothers in Ghana. Chen et al. [32] show that Taiwan’s NHI has significantly increased the utilization of both outpatient and inpatient care among the elderly. Zhang et al. [33] show that participating in medical insurance has a positive effect on the utilization of health care by migrant workers in China, while Mensah et al. [34] find a positive impact of the Ghanaian NHIS on access to health care. Agbanyo [23] and Agbanyo and Peprah [20] also find the same results. In the light of these results, the Togolese government should implement policies aimed at increasing the coverage of the MHI scheme in the country. An increase in the coverage of the MHI scheme is likely to improve the general health of the population, which may lead to an improvement in productivity. In this regard, the law on universal health insurance which was passed in October 2021 by the National Assembly is an opportunity. The results reveal a high rate of non-utilization of healthcare professionals in the case of illness, even among individuals with MHI coverage. Although a sizable portion of insured individuals did not see any health care professionals in the case of illness, the MHI is positively correlated with the likelihood of seeing health care professionals in the event of illness. This correlation may be explained by the fact that more insured individuals than uninsured individuals see a health care professional in the case of illness. The lack of money and self-medication were the most important reasons for non-utilization of health care in the case of illness. Although fewer insured individuals than uninsured individuals do not see a health care professional in the case of illness, the fact that a sizable proportion of insured individuals do not see a health care professional in the case of illness could reflect a perceived poor quality of health care. In fact, compared to 15% of uninsured people, 18% of insured people are dissatisfied with the care they received during their consultation. Similarly, compared to 7% of those without insurance, almost 15% of those with insurance reported receiving unsatisfactory service. These results can be explained by the fact that care providers devote more time and energy to uninsured patients, who pay the costs of health care directly, than to those with insurance, which requires cumbersome administrative procedures before reimbursement [13]. The government should therefore take action to raise the standard of treatment provided to insured patients in health care centers. Moreover, the poverty rate is high in the country [11]. The 20% of medical expenses that must be paid by the insured could be a great burden for low-income insureds. In the event of illness, this may discourage them from seeking treatment at formal health centers. These results corroborate those of Atake and Amendah [13], who showed that a significant proportion of the insured face catastrophic health expenses in Togo. The government should therefore consider waiving medical fees for low-income policyholders, as suggested by Atake and Amendah [13].

As expected, the findings show that, being hospitalized in the last 12 months preceding the survey is positively correlated with the likelihood of seeing formal health care professionals, rather than not seeing any health care professional in the case of illness. Hospitalization prior to the survey may be a good proxy for the presence of a household member with a chronic disease in the household. This result also corroborates Andersen’s behavioral model [28–30] and is consistent with those of Atake [12], who finds that the presence of a household member with a chronic disease has a positive impact on health care utilization in Togo. People with chronic diseases make intensive use of health care due to the fact that their health is disproportionately complex and difficult for them to manage [12, 35].

All else being equal, we find that the size of the household reduces the likelihood of seeing a specialist in the event of illness, rather than not seeing any health care professional at all. This result is in contradiction to those of Atake [12], who finds that the increase in the size of the household significantly increases the likelihood of seeking health care in Togo. Similarly, Bolduc et al. [36] find that in Benin, having a large family increases the probability of resorting to public and private health centers rather than using self-medication. The fact that our results show a negative effect of the size of the household on the likelihood of seeing formal health care professionals in the case of illness may be explained by the positive relationship between poverty and the size of the household [37, 38]. Anyanwu [37] finds that a one-person household negatively and significantly reduces poverty, while the addition of members to the household progressively increases the likelihood of being poor in Nigeria. Libois and Somville [38] also find a positive relationship between the size of the household and poverty in Nepal. The size of a household could imply a significant economic burden on families [12]. Thus, as the size of a household increases, the likelihood of seeing a formal health care professional in the case of illness decreases, given that the economic burden of illness becomes increasingly heavy, especially for households without health insurance. The fact that our results are in contradiction to those of [12] may be explained by the fact that Atake [12] focuses on insured households, while our sample includes both insured and uninsured individuals.

The results show that the probability of seeing a specialist physician is statistically and significantly higher among urban residents than among their rural counterparts. The same is true for the likelihood of seeing a general physician. Similarly, living in the Grand Lomé region increases the likelihood of seeing a specialist physician compared to living in the Maritime region. When compared to the rest of the country, the likelihood of seeing a general practitioner is statistically and significantly higher in the Maritime region. These results could be explained by the disparities in the availability of health care facilities between rural and urban areas [39], on the one hand, and regional disparities in terms of poverty, on the other [11]. Supply-side interventions targeting rural areas should be an effective way to reduce the disparity between urban and rural areas. When waiving medical costs for low-income policyholders, the Togolese government ought to focus on the regions with the worst economic conditions. These interventions should be essential to ensure that no one is left behind.

We find that the gender of the household head has no statistically significant effect on the likelihood of seeing formal health care professionals. This result could be explained by the effectiveness of the National Policy of Equity and Gender Equality (PNEEG) implemented by the Togolese government over the period 2013–2017 [40].

### Limitation

This study has some limitations. Firstly, the data used to measure the various indicators were declared by the respondents themselves and cannot be verified using administrative sources. Secondly, given the availability of data, we did not take into account the distance to the nearest health center in our econometric analysis. Yet, this variable has been identified as an important determinant of health care utilization [12]. However, we hope that by including transport goods, the bias is reduced. Furthermore, our findings indicate that only 1.1% of respondents cited distance as a major barrier to seeking medical attention for a sickness within the previous 30 days. But some caution is required when interpreting the findings.

## Conclusion

This study analyzes the relation between MHI and health care utilization in Togo. Using a multinomial logistic regression, the main results indicate a positive correlation between the MHI scheme and health care utilization in Togo. Factors such as the household’s wealth, the size of the household, the region, and the residence area are statistically and significantly associated with the likelihood of seeing health care professionals in the case of illness. Furthermore, the results reveal a high rate of non-utilization of healthcare professionals in the case of illness, even among individuals with MHI coverage. The lack of money and self-medication were the most important reasons for non-utilization of health care in the case of illness. Several policy implications emerge from the study.

- ✓ The Togolese government should implement policies aimed at increasing the coverage of the MHI scheme in the country.
- ✓ The government should take action to raise the standard of treatment provided to insured patients in health care centers.
- ✓ The government should consider waiving medical fees for low-income policyholders.
- ✓ When waiving medical costs for low-income policyholders, the Togolese government should focus on the regions with the worst economic conditions. These interventions should be essential to ensure that no one is left behind.
- ✓ The difference between urban and rural communities should be reduced through supply-side policies that focus on rural areas.

## Data Availability

The data supporting the conclusions of this article are publicly available from the World bank, upon registration at https://microdata.worldbank.org/index.php/catalog/4298.

## Abbreviations

CBHI: Community-Based Health Insurance
CHE: Current Health Expenditure
EHCVM: Harmonized Survey on Living Conditions of Households
INAM: National Health Insurance Institute
INSEED: National Institute of Statistics, Economic Studies, and Demography
ISW: Informal Sector Workers
LR: Likelihood Ratio
MHI: Mandatory Health Insurance
NHI: National Health Insurance
NHIS: National Health Insurance Scheme
OOP: Out-of-pocket Payments
PNDS: National Health Development Plan
PNEEG: National Policy of Equity and Gender Equality
VHI: Voluntary Health Insurance
VIF: Variance Inflation Factors
WAEMU: West African Economic and Monetary Union
WTP: Willingness-to-pay
